# The Rectifying Contact of Hydrated Different Size YSZ Nanoparticles for Advanced Electronics

**DOI:** 10.3390/nano12244493

**Published:** 2022-12-19

**Authors:** Alexander S. Doroshkevich, Anna S. Zakharova, Boris L. Oksengendler, Andriy I. Lyubchyk, Sergiy I. Lyubchyk, Svitlana B. Lyubchyk, Alisa A. Tatarinova, Andriy K. Kirillov, Tatyana A. Vasilenko, Oksana O. Gorban, Viktor I. Bodnarchuk, Nadejda N. Nikiforova, Elena A. Zakharova, Maria Balasoiu, Diana M. Mardare, Carmen Mita, Anca Stanculescu, Matlab N. Mirzayev, Asif A. Nabiyev, Evgeni P. Popov, Le Hong Khiem, Alexander A. Donkov, Vesna Teofilović, Bozena Jasinska, Dan Chicea, Tatyana Ye. Konstantinova

**Affiliations:** 1 Department of Material Science, Donetsk Institute for Physics and Engineering Named after O.O. Galkin, 03028 Kiev, Ukraine; 2 Joint Institute for Nuclear Research, 141980 Dubna, Russia; 3 Faculty of Physics, M.V. Lomonosov Moscow State University, 119991 Moscow, Russia; 4 Ion-Plasma and Laser Technologies Institute after U. Arifov, Tashkent 100125, Uzbekistan; 5 Nanotechcenter LLC, 03680 Kyiv, Ukraine; 6 Research Centre in Industrial Engineering Management and Sustainability, Lusófona University, 1749-024 Lisbon, Portugal; 7 REQUIMTE, Faculdade de Ciências e Tecnologia, Universidade Nova de Lisboa, 2829-516 Caparica, Portugal; 8 The Research Center for Geomechanics and Mining Problems, Saint-Petersburg Mining University, 199106 St.-Petersburg, Russia; 9 Institute of Oil Refining and Petrochemistry FSBEI of HE USPTU, 450064 Salavat, Russia; 10 Nuclear Physics Department, Horia Hulubei National Institute for R&D in Physics and Nuclear Engineering (IFIN-HH), 077125 Bucharest, Romania; 11 Faculty of Physics, “Alexandru Ioan Cuza” University of Iasi, 700506 Iasi, Romania; 12 Faculty of Chemistry, “Alexandru Ioan Cuza” University of Iasi, 700506 Iasi, Romania; 13 Optical Processes in Nanostructured Materials Laboratory, National Institute of Materials Physics, 077125 Magurele, Romania; 14 Institute of Radiation Problems, Azerbaijan National Academy of Sciences, Baku AZ1143, Azerbaijan; 15 Institute of Solid-State Physics, Bulgarian Academy of Sciences, 1784 Sofia, Bulgaria; 16 Institute for Nuclear Research and Nuclear Energy, Bulgarian Academy of Sciences, 1784 Sofia, Bulgaria; 17 Graduate University of Science and Technology, Vietnam Academy of Science and Technology, Hanoi 11355, Vietnam; 18 Institute of Physics, Vietnam Academy of Science and Technology, Hanoi 11108, Vietnam; 19 Faculty of Technology, University of Novi Sad, 21000 Novi Sad, Serbia; 20 Institute of Physics, Maria Curie-Skłodowska University, 20-031 Lublin, Poland; 21 Department of Environmental Sciences, Physics Group, “Lucian Blaga” University of Sibiu, 550012 Sibiu, Romania

**Keywords:** YSZ nanoparticles, dimensional effects, advanced electronics, breakthrough critical technologies

## Abstract

The paper considers the new effects of the nanoscale state of matter, which open up prospects for the development of electronic devices using new physical principles. The contacts of chemically homogeneous nanoparticles of yttrium-stabilized zirconium oxide (ZrO_2_—*x* mol% Y_2_O_3_, *x* = 0, 3, 4, 8; YSZ) with different sizes of 7.5 nm and 9 nm; 7.5 nm and 11 nm; and 7.5 nm and 14 nm, respectively, was studied on direct current using nanostructured objects in the form of compacts obtained by high-hydrostatic pressure (HP-compacts of 300MPa). A unique size effect of the nonlinear (rectifying-type contact) dependence of the electrical properties (in the region *U* < 2.5 V, *I* ≤ 2.7 mA) of the contact of different-sized YSZ nanoparticles of the same chemical composition is revealed, which indicates the possibility of creating semiconductor structures of a new type (homogeneous electronics). The electronic structure of the near-surface regions of nanoparticles of studied oxide materials and the possibility of obtaining specifically rectifying properties of the contacts were studied theoretically. Models of surface states of the Tamm-type are constructed considering the Coulomb long-range action. The discovered energy variance and its dependence on the curvature of the surface of nanoparticles made it possible to study the conditions for the formation of a contact potential difference in cases of nanoparticles of the same radius (synergistic effect), different radii (doped and undoped variants), as well as to discover the possibility of describing a group of powder particles within the Anderson model. The determined effect makes it possible to solve the problem of diffusion instability of semiconductor heterojunctions and opens up prospects for creating electronic devices with a fundamentally new level of properties for use in various fields of the economy and breakthrough critical technologies.

## 1. Introduction

Diffusion instability of classical semiconductor structures [1], leading to inevitable failures of electronic equipment, is a serious scientific and technical problem of modern critical technologies [2].

There are a number of theoretical works that discuss possible ways to solve this problem [3,4,5,6,7], however, so far it has not been possible to find possible options for the practical implementation of devices devoid of this drawback. According to the authors of this work, the problem can be solved by using new physical principles in the creation of functional semiconductor structures, in particular, the effects of the low-dimensional state of matter [8]. The concept of the work is the replacement of semiconductor heterojunctions, in which excess charge carriers are formed by implanting a non-valent impurity with functional junctions, in which excess charge carriers are formed as a result of size effects of band structure distortion (nanoscale objects).

The nanopowder system based on ZrO_2_ is characterized by dimensional effects of an adsorption nature and therefore is interesting for practical application. In particular, the previously determined effects, such as adsorption-induced electrical conductivity [9], localization and transport of charge carriers to electrodes [10,11], accumulation of electric charge [12,13], and the size effect of the appearance of periodic current pulsations in compacts of zirconium dioxide nanopowder during a discharge after exposure to a constant current [14] against the background of a finite electrical conductivity (ionic conductivity) of the system, indicate the presence of adsorption-induced interphase electron exchange and its (system) electrical continuity. Therefore, it is possible to generate and remove nonequilibrium charge carriers from the functional layer, which is formed upon contact of two nanoparticles of the same chemical composition, but with different surface geometry (particle size) due to the size effect of energy band distortion. Therefore, a hydrated nanopowder system based on ZrO_2_ can be used to implement a new type of functional transition. Experimental and theoretical study of the electrical properties of the contact of hydrated nanopowder YSZ systems is the aim of this work.

## 2. Materials and Methods

**Figure 1:**
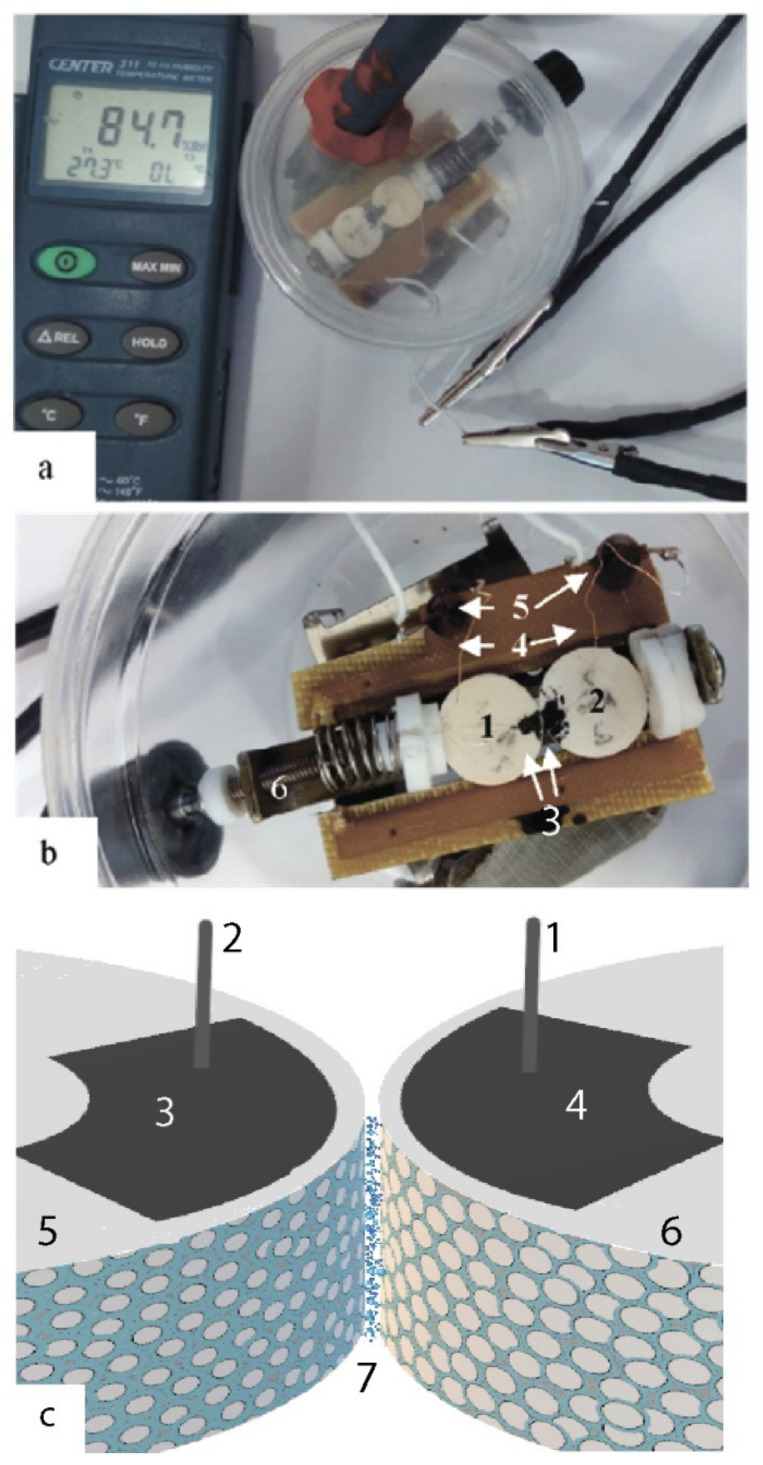
External view of the sample chamber (**a**), and the sample holder (**b**), where 1, 2—the tablets under study; 3—contact pads; 4—current collectors; 5—fixing racks; 6—mechanical spring-loaded clamp with adjustable force. The scheme of the experiment (**c**), where 1—anode; 2—cathode; 3, 4—contact pads; 5—a tablet of a powder with a smaller particle size, 6—a tablet of a powder with a large particle size, 7—a hydration layer.

The sample is a contact of two compacts of nanopowders of the composition ZrO_2_—3 mol% Y_2_O_3_ with a particle size of 7.5 and *x* nm, where *x* = 7.5 nm (400); 10 nm (500); 12 nm (600); 14 nm (700) [15] in the tablet form Figure 1a.

To obtain nanopowders, the chemical technology of co-deposition with the use of physical effects was used [16]. First, hydrated zirconium hydroxide was obtained by precipitation from chloride raw materials. After dehydration in a special microwave oven (*T* = 120 °C, *t* = 0.4 h), the amorphous powder was subjected to crystallization annealing for 2 h at 400 °C for the object 5 (Figure 1) and at 500 °C for the object 6, (Figure 1) contacting object for 2 h. Additionally, the annealing time of 2 h was for temperatures of 600 °C (particle size 12 nm) and 700 °C (particle size 14 nm).

The compacts were obtained from powders of the composition ZrO_2_—3 mol% Y_2_O_3_ by uniaxial pressing (*P_comp_* = 40 MPa) in the form of tablets with a diameter of 20 mm and a height of 3.2–3.3 mm (weighed amount *m* = 1.2 g), then compaction was made with high hydrostatic pressure (HHP, 300 MPa) in the installation type UVD-2 (DonPhTI). The HHP diameter of the compacts decreased to 16mm on average, and the height decreased to 2 mm.

The sample chamber was a closed container with a volume of 350 mL with the atmospheric humidity controlled using salts [17] (Figure 1a).

The sample under study was a contact of two tablets of nanopowders with the composition ZrO_2_—3 mol%Y_2_O_3_ with particle sizes of 7.5 and 10 nm (Figure 1c).

Current collectors made of tinned copper wire 0.01 mm in diameter were glued to the ends of the tablets (1, 2) near one of the edges using a conductive varnish based on nickel powder (MASTIX) specifications 2262-018-90192380-2011, Figure 1b. Conductive areas (3) formed during the drying of conductive varnish, to which the current collectors (4) were glued, served as electrodes. The area of the electrodes was about 3 mm^2^. Current collectors were fixed on special fixing racks (5). From the racks, wires were leading directly through the walls of the chamber. The positive electrode “+” was connected to compact 400, and the negative electrode “−” to compact 500/600/700. The tablets were brought into contact by a special mechanical spring-loaded clamp (6) with an adjustable force (about 10 N). The shape of the contacting surfaces of the tablets was linear-elliptical.

The sample chamber was a closed container with a volume of 350 mL with atmospheric humidity controlled using KCl (60%), NaCl (76%), and NaBr (86%) salts. 

The spatial and structural organization of the samples was investigated by transmission (TEM) and scanning (SEM) electron microscopy using JEM 200A and JSM640LV (JEOL, Japan) instruments correspondingly.

Voltammograms (*V*-*I* characteristics) were obtained in a linear sweep mode (from −6 V to +6 V) on an R-20 device (“Elinns”) in a moisture saturation mode at three points (86, 76, and 60%). The experiment was carried out in 3-fold repetition. The positive electrode was placed on a powder tablet with a smaller nanoparticle size. The sequence of measuring electrical parameters is as follows: from maximum (humidity = 86%) to minimum (humidity = 60%). The saturation times of the samples at each humidity are at least 2 h (until the adsorption equilibrium was established in the system: working volume—salt—sample) with an accuracy of 5%.

## 3. Results

### 3.1. Morphology of the Studied Objects

**Figure 2:**
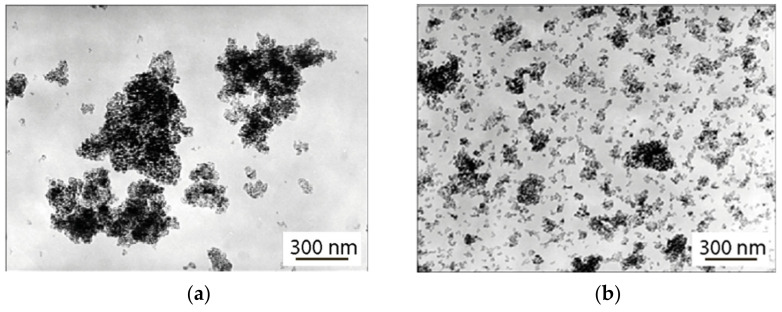
TEM images of nanopowders of the composition ZrO_2_—3% mol Y_2_O_3_, obtained at annealing temperatures of 400 °C, during 2 h. (**a**), and 500 °C during 2 h (**b**).

TEM images of nanopowders of the composition ZrO_2_—3 mol% Y_2_O_3_ obtained at calcination temperatures of 400 °C and 500 °C are shown in Figure 2. The figure shows that nanoparticles form relatively loose aggregates up to 1 nm in size in the case of a powder obtained at 400 °C, and no more than 300 nm in the case of a powder obtained at 500 °C. Therefore, the 9 nm powder is better distributed over the substrate than the 7.5 nm powder. This indicates a lesser cohesion of particles in it. Tablets made of nanopowders have approximately the same density (approximately 3 g/cm^3^) and porosity of about 50% [18].

**Figure 3:**
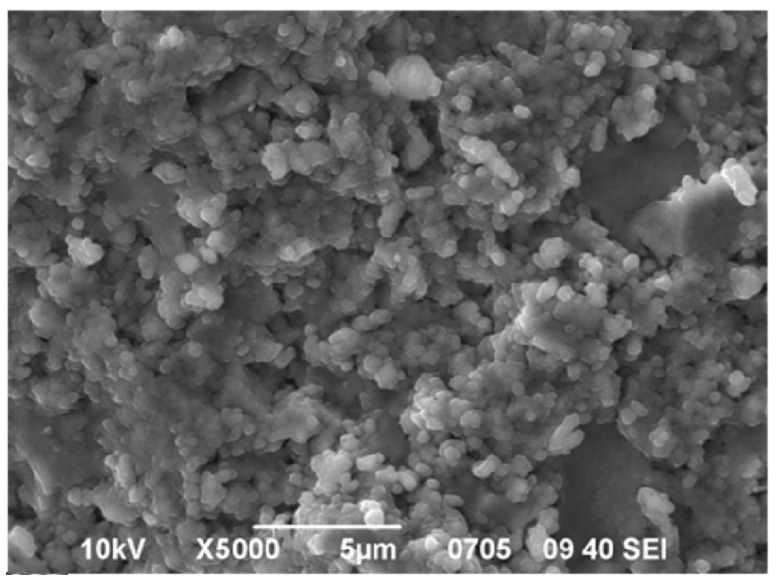
A typical SEM image of a fracture in a nanopowder compact with the composition ZrO_2_—3% mol Y_2_O_3_ (The nanopowder was obtained at an annealing temperature of 400 °C).

The SEM image, Figure 3, shows the fracture morphology of the compact. A significant number of relatively large (about 1 µm) pores can be seen, obviously serving as channels for moisture penetration into the bulk of the compact material. Consequently, moisture relatively easily penetrates the bulk of the samples and the limit time factor is its adsorption by the interface [19].

To compensate for the valence-unsaturated electron orbitals of surface atoms—chemically active centers, ZrO_2_ nanoparticles adsorb electrically neutral molecules, mainly water, from the gas atmosphere, [20]. When interacting with a surface, a molecules are polarized (in the case of physical contact) or exchange electrons (with dissociation) if the interaction is chemical. Thus, as a result of the implementation of adsorption processes, the surface of nanoparticles is covered with an electrically conductive hydration shell [21,22], due to which the continuity of the electrical properties of the compact material is realized. Charge transfer over the surface of nanoparticles is carried out by means of protons [23,24]. It should be noted that the quantitative composition of the adsorption shell of nanoparticles depends on the amount of moisture in the atmosphere, so as a consequence, the electrical conductivity of the nanopowder system dynamically changes with a change in atmospheric humidity.

### 3.2. Contact of Different Sized Nanoparticles

**Figure 4:**
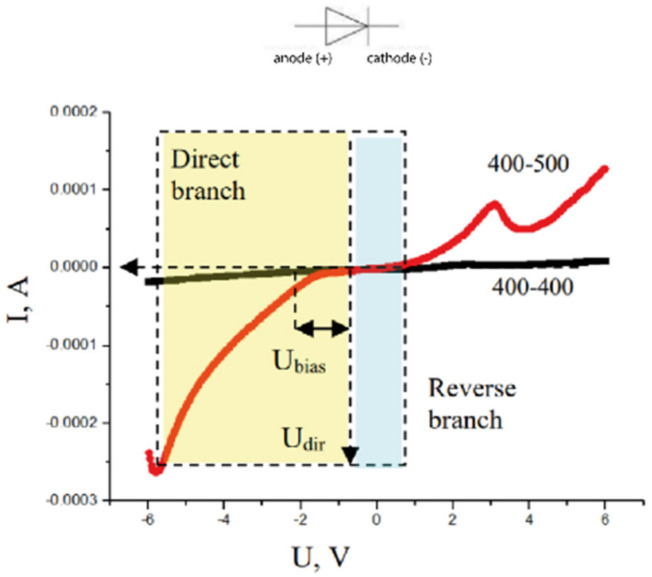
A *V*-*I* curves of contacts of chemically identical samples (ZrO_2_—3 mol% Y_2_O_3_) with identical size of particles (curve 400–400) and samples with different size of particles (curve 400–500) at humidity (85%).

Figure 4 shows the current versus voltage dependences obtained at an atmospheric humidity of 85%.

It can be seen that the dependence of the current on the voltage for the contact of systems with the same particle size (curve 400–400) on a given scale has a linear character, while a similar dependence for the contact of systems with different sizes of nanoparticles (curve 400–500) has a pronounced “diode” character in the voltage range from −5 to +2 V. The forward branch of the semiconductor junction with the used arrangement of the electrodes is in the fourth octant, and the reverse branch is in the first, see Figure 4. The linear nature of the heterojunction between compacts with the same particle size (400–400) indicates a relatively low current (up to 20 μA) through the contact at the specified humidity. The contact of compacts of different-sized particles at the same moisture content demonstrates an order of magnitude higher (up to 250 μA) current.

The working range of the obtained heterojunction is about 5 V—forward branch, 2 V—reverse. After exceeding these values, the loss of its straightening properties occurs. The curve is offset from zero to negative values. It can be assumed that the presence of a bias voltage of −2 V is due to the polarization of the contact or electrodes.

### 3.3. Influence of Humidity, Particle Size Difference and the Amount of Impurity in the Composition of the Nanoparticle Material on the Electrical Properties of the Contact

The thickness and conductivity of the hydration layer depends on the particle size [21] and the atmospheric humidity. The hydration shell is continuous under normal physical conditions for systems with a particle size of up to 9 nm (annealing temperature of 500 °C). Above 14 nm (annealing temperatures above 700 °C), the hydration shell has an “island”—type character [25]. Conductivity in the system occurs when the hydration shells of individual nanoparticles overlap; therefore, there is a percolation threshold for electrical properties.

**Figure 5:**
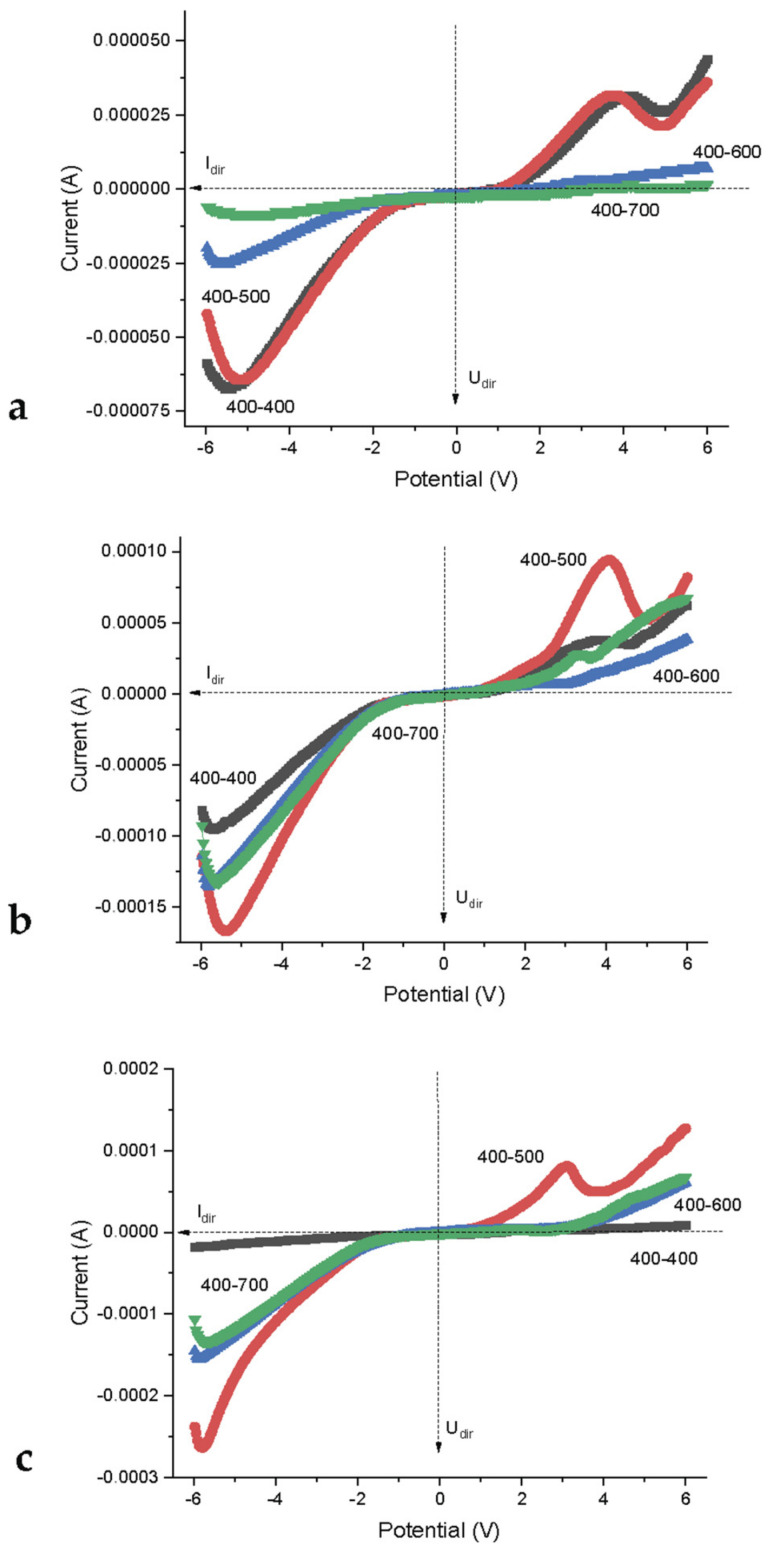
Families of curves *A*-*I* of contacts of chemically identical samples (ZrO_2_-3 mol% Y_2_O_3_) with identical size of particles 7.5 nm (curves 400–400) and samples with different size of particles: 7.5 nm–9 nm (curves 400–500); 7.5 nm–11 nm (curves 400-600); 7.5–14 nm (curves 400–700) at relative atmospheric humidity 60% (**a**), 76% (**b**) and 86% (**c**).

In Figure 5a, *V*-*I* characteristic dependences of four contacts formed by chemically homogeneous nanostructured objects differing in the sizes of nanoparticles after hydration in an atmosphere with different concentrations of moisture vapor are presented on an optimized scale. At lower humidity, the system is beyond the percolation threshold in a nonconducting state [26] and cannot be investigated by the measuring instruments used in this work.

It can be seen from Figure 5a that at low humidity, the *V*-*I* characteristics of contacts 400–600 and 400–700 differ significantly from the *V*-*I* characteristics of contacts 400–400 and 400–500 by a small value of operating currents (Table 1). The characteristic features in the range of voltage values of −5 V and +4 V on the curves are also very weakly expressed. It can be assumed (Figure 5a) that in contrast to mutually well-hydrated low-dimensional systems (400–400 and 400–500), systems containing larger particles (11 and 14 nm for powders obtained, respectively, at 600 °C and 700 °C) at a humidity of 60% are close to the threshold of electrical percolation. Contacts 400–600 and 400–700 have a slightly increasing linear characteristic. Systems 400–400 and 400–500 exhibit the properties of a “false” compound at 60% humidity [27], in which Ohm’s law is violated due to the electrophysical transient processes.

At a humidity of 76% (Figure 5b) contacts 400–400 and 400–500 begin to change their behavior significantly: *V-I* contacts 400–400 begin to level out, and *I-V* contacts 400–500, on the contrary, bend and “turn” into a rectifying one. Contacts 400–600 and 400–700 acquire the *I-V* characteristics of the rectifier type. The trend persists with a further increase in atmospheric humidity (saturated vapor pressure). At 85%, their limit characteristics differ from those for a 400–500 contact by half the limit forward current (1.4–1.5 mA and 2.7 mA), and almost twice as large as the maximum reverse voltage (1.8 V, and 3.5 V, Table 1).

The peak in the +3 V region is probably due to the water dissociation processes. The peak at *U* = −6 − −5.5 V on the forward branch is probably a concentration limitation on the majority of charge carriers. It can be concluded that the majority of charge carriers in this system are positively charged.

**Figure 6:**
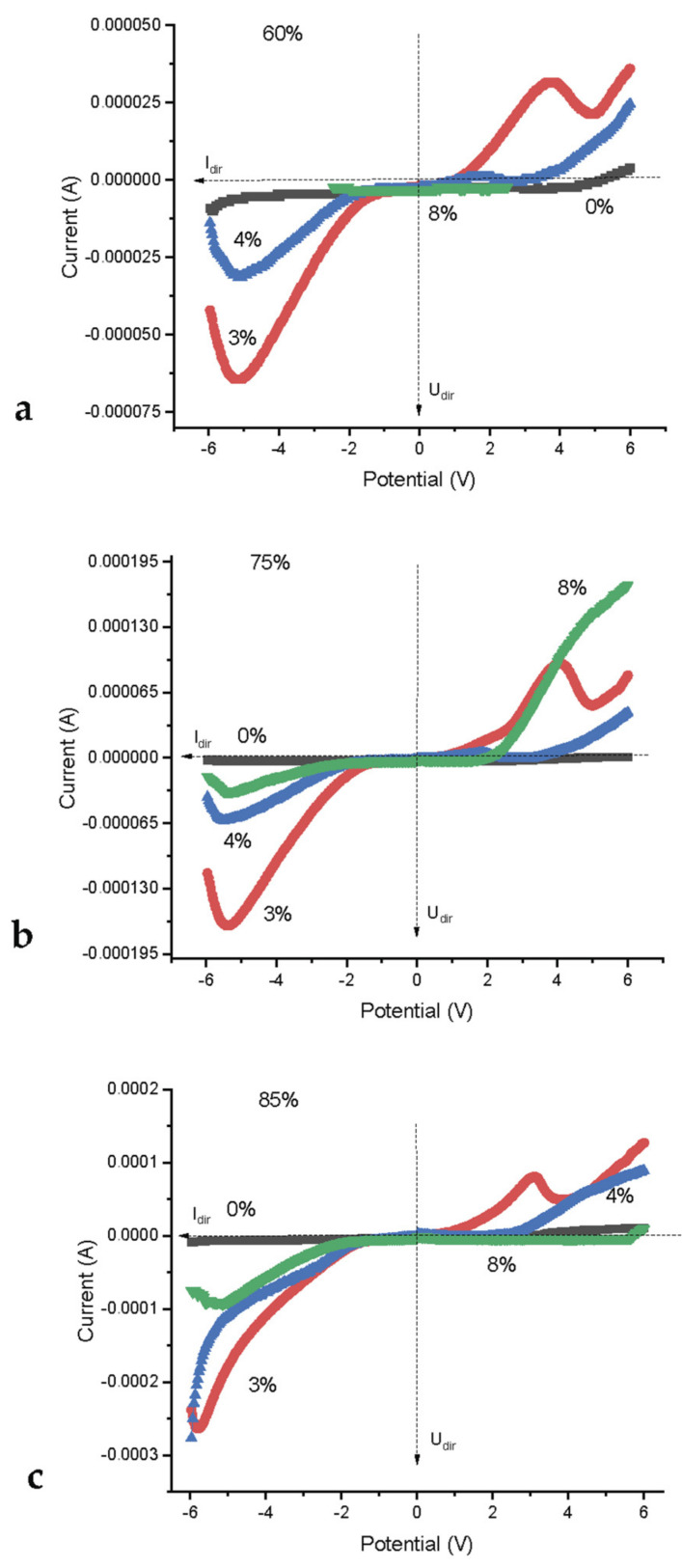
Dependence of current on voltage at contacts 400–600 based on ZrO_2_-*x* mol% Y_2_O_3_ nanopowders with yttrium content *x* = 0, 3, 4, 8mol% at humidity 60% (**a**), 75% (**b**) and 85% (**c**).

**Figure 7:**
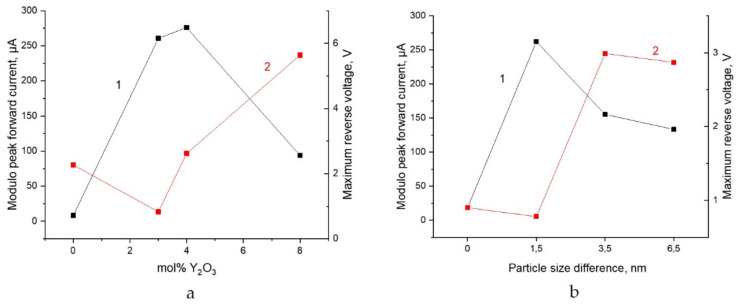
Dependence of the limiting values of forward current (curve 1) and reverse voltage (curve 2) at a humidity of 85% on the content of yttrium nanoparticles in the composition of the material (**a**) and the difference in particle diameters (**b**).

Figure 6 shows the current versus voltage dependences for systems differing in the concentration of the alloying component (Y_2_O_3_). At all humidity concentrations, there is a clear dependence of the current amplitude on the impurity concentration. A system without an alloying component undergoes minimal changes in the electric field (less than 5–10% of the same value for systems with 3 and 4 mol% Y_2_O_3_) for any humidity levels. Increasing the impurity concentration by more than 3 mol% leads to an increase the current amplitude or the reverse voltage modulus on the electrodes. i.e., as in the case of size dispersion, Figure 5, with variations in the impurity concentration in the composition of the material of contacting nanoparticles, the *V*-*I* dependences have an extreme character (Figure 6). It can be seen that the value of the limit current (the direct branch of the *V*-*I* characteristic (third quadrant), reaches the maximum value on all spectra, (Figure 6b) at a concentration of Y_2_O_3_ 3 mol% and then sharply (more than twice) decreases with an increase in the impurity concentration by another 1 mol%. An increase in the impurity concentration by more than two times—up to 8%, leads to a proportional decrease (approximately by another two times) in the amplitude of the forward branch current, but an increase in the length of the rectilinear section of the reverse branch. The latter effect depends on the concentration of moisture in the system and is most pronounced when the vapor concentration corresponds to a relative humidity of 85% (Figure 6c). It should be noted that under the same conditions, the maximum effect is achieved in terms of the value of the forward current in the case of a system with 3 mol% Y_2_O_3_. Thus, the concentration of humidity vapor corresponding to 85%, as in the case of the dimensional dispersion (Figure 5), ensures that the system reaches the maximum limit parameters corresponding to a particular concentration of impurities (Table 2). The behavior of the limit parameters of the system depending on the impurity concentration and the difference in the sizes of nanoparticles at a humidity of 85% is shown in Figure 7.

A decrease in the concentration of humidity in the system leads to a decrease in the level of electrical characteristics of the contact, which characterize its rectifying properties. In particular, both the level of limit characteristics and the nature of the dependences decrease, in particular, when the contact passes from the state of a rectifying contact with the most pronounced asymmetry of the *V*-*I* curve in the first and third quadrants to the state of the so-called “false connection” (Figure 6a).

### 3.4. Relationship between Structural and Electrical Properties of the System

It can be seen (Table 2, Figure 7a) that the maximum forward current values are observed at an impurity content of 3–4% mol Y_2_O_3_. Approximately in the same range of 2–3% mol Y_2_O_3_, there is a minimum of the values of the limit reverse voltage. Consequently, several competing processes contribute to the conductivity, presumably due to “size-” and “structural-” factors. The 2–4% region in the ZrO_2_—Y_2_O_3_ phase diagram is characterized by the maximum structural metastability. In the case of nanoparticles, phase transformations (β-α) are reversible [28], therefore, the emission of free charge carriers in the system is most likely due to the electrically stimulated phase transformations under hydration conditions. At a dopant concentration of more than 3% mol, the thermodynamic stabilization of the system (β and γ-phases) occurs, while the reverse voltage increases monotonically, and the forward current decreases monotonically. A similar dependence of the limit parameters is observed on the difference in particle sizes (Figure 7b). Only the size factor does not lead to hysteresis. It should be noted that the effect practically does not manifest itself upon contact of monodisperse particles. The reverse voltage minimum (minimum field) and the forward current maximum are observed at a diameter difference of *Δd* = 1.5 nm. The subsequent increase in the diameter difference *Δd* leads to a proportional increase and stabilization (at the level of 3 V at *Δd =* 5.5 nm) of the reverse voltage with a monotonous decrease in the forward branch current.

The calculated electrical power of the resulting device does not depend significantly on the difference in the sizes of the contacting nanoparticles and, other things being equal, is 4.86 mW for the 400–500 system and 4.9 mW for the 400–600 and 400–700 systems. 

The experiment proves the difference in some electrical and electronic properties of the generalized surface of different-sized particles.

## 4. Interpretation of the Effect

### 4.1. The Nature of the Electronic Component in Ionic Nanocrystals

According to [29], adsorption significantly changes the electronic structure and physical properties of the material of nanoparticles; in particular, it imposes a spectrum of local levels of adsorbates (surface states of impurity type) on the energy spectrum of states of non-adsorption origin. This, first of all, leads to recharge of the surface and localization in the near-surface region of nanoparticles of charge carriers. In the acceptor nature of the adsorption electronic state, the surface is charged negatively in a thin layer (Debye screening length) *L = f (ε, T, n)* near the surface, and a p-type space charge region (SCR) is formed [30]. This is shown in an increase in the conductivity of the near-surface layer of nanoparticles and the overall conductivity of the system [22], and in the appearance of a relatively high electrical capacity in the nanopowder system and the possibility of charge exchange with the external environment [31].

The assumption of adsorption-induced localization of electron-type charge carriers (formation of “electron gas”) in the near-surface region of nanoparticles is confirmed by molecular dynamics (MD) calculations. In [32], MD methods have shown a sharp increase in the density of localized electronic states in the band gap (BG) during the adsorption of moisture on the surface of the ZrO_2_—Y_2_O_3_ crystal.

The magnitude of the surface potential depends on the number of molecules adsorbed on the surface and on the size and shape of the particle surface itself. Surface geometry leads to a significant modification of Tamm states. Thus, we can distinguish the following phenomena: (1) the stretching of Tamm’s orbitals outside the particle with a decrease in its size [33] (the formation of chemically uncompensated valence orbitals—“dangling bonds” capable of “capturing” incident molecules from the gas phase); (2) an increase in the Coulson free valence index of Tamm orbitals with an increase in the curvature of the surface [34]; and (3) the shift of a pair of levels of Tamm states to the middle of the band gap as the curvature of the surface of nanoparticles in ionic crystals increases (decrease in the effective band gap) [35]—a decrease in the electron localization energy when the energy bands are curved as a result of surface charging. For example, for adsorbates, there is a “collapse” of the forbidden zone during the adsorption of water molecules [12,36]. According to [37], the magnitude of the field between the volume and the surface of nanoparticles (surface potential) depends on the particle size d in the range −1 ≤ *d*/2*L_ef_* ≤ −2.2 due to the overlap of the SCR from opposite surfaces.

### 4.2. Electronic Mechanism

The proposed mechanism is based on the assumptions about the confinement of electronic excitations and the commensurability of the number of surface and bulk electronic states [38] inherent in nanoscale objects. The zone model of Tamm (1932) modified with the use of fractal geometry makes it possible to consider the form factor of the surface of nanosized objects and can be used to describe the electro-surface effects considered in this work [34,39].

**Figure 8:**
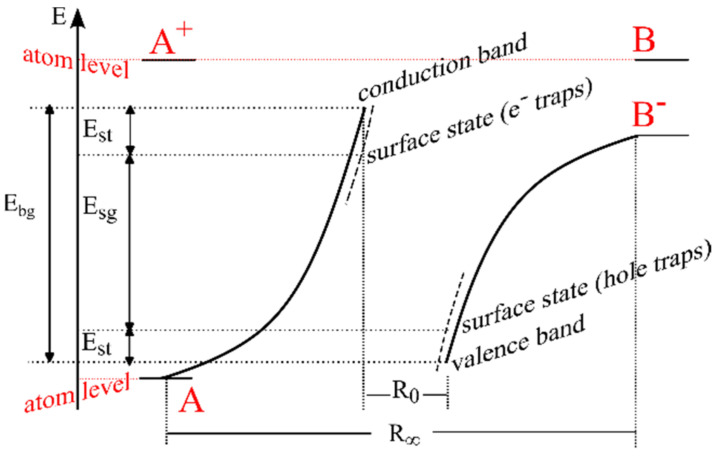
Classical energy diagram of Madelung-Seitz in ionic crystals of type AB. Adapted from [40].

The classic Madelung-Seitz diagram [40] shows the energy levels as a function of the interatomic distance *R* in ionic crystals of type AB (NaCl) with one limited surface, Figure 8. According to Seitz [33], the band gap energy is directly related to the Madelung energy, both in the volume and on the surface.

**Figure 9:**
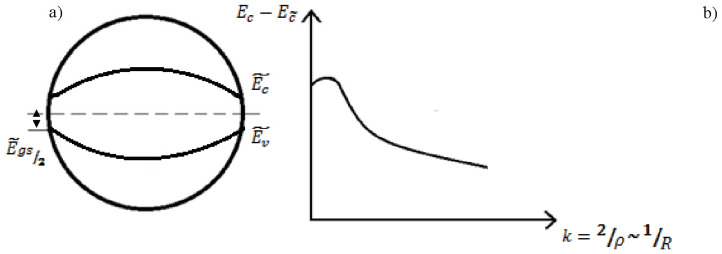
Schematic representation of the energy level of an impurity of a spherical nanoparticle (**a**) and the dependence of its energy on the surface curvature *R* in a two-zone model (**b**).

Indeed, Figure 8, which reproduces Seitz’s reasoning, shows the origin of the solid-state electronic spectrum from the electronic spectrum of NaCl atoms. Obviously, the width of the band gap in the crystal volume is a function of the size/shape of the crystal (conditioned by the Madelung constant in the bulk (*L_b_*) as well as on the surface (*L_s_*)). It is clear that *L_s_ < L_b_*, since the Madelung sums for a surface ion are approximately half as large as the volume sums. Simple physics makes it possible to understand that, in this case, “two Tamm levels” on the surface have a very simple genesis—these are the levels of the edges of the conduction band and the valence band tightened to the middle of the band gap, see Figure 9a.

Thus, the energy distance between two levels near the surface (donor and acceptor) is less than the band gap in the bulk.

According to [34,39], for an ion located at the top of a convexity in the case of a convex surface, when calculating the Madelung energy, new additional regions of the crystal with ions located in them will no longer be enough. This immediately leads to a further shift of the “two Tamm levels” towards the middle of the forbidden band in Figure 9. The dependence of the band gap on a curved surface on the degree of its curvature has the form $k=\frac{2}{\rho }$, where *ρ* is the radius of curvature, and with increasing curvature, the surface band gap decreases, $\frac{d}{dR}E_{gs}>0$, Figure 9b.

In the case of contact of two spherical nanoparticles with *R*_1_ > *R*_2_ with an ionic bond Figure 1c, there is a heterojunction of identical materials, but of different particle radii. With a sufficient degree of similarity, we can speak here as about the contact of two graded-gap semiconductors, but in order to reveal the most important property of such a structure—the contact potential difference, it is possible to ignore the variation-gap in the first approximation. It is easy to show that the confinement of electron–hole excitations in nanoparticles of different radii leads to a difference in the ionization potentials and electron affinity energies in these particles with different radii, which immediately destroys the equality of the Fermi level in these two samples even in the absence of alloying [41].

If there is a deep level from a certain defect (alloying impurity) in the band gaps on the surfaces of the contacting particles, provided that it does not coincide with the Fermi levels due to the smallness of these band gaps, the wave function of such an electronic state on the defect is a kind of superposition of the eigenstate of the defect, states conduction and valence bands (Keldysh 1963) [42,43]:

(1) $$\psi_{i}\left(z\right)=\psi_{0}\left(z\right)+\int_{cz}c\left(\varepsilon \right)\varphi_{k}\left(\varepsilon \right)d\varepsilon +\int_{vz}\tilde{c}\left(\varepsilon \right)\tilde{\varphi_{k}}d\varepsilon$$

where *cz*—conduction zone; *vz*—valence zone.

Here, the coefficients c(ε) and $\tilde{c}\left(\varepsilon \right)$ give weight factors for the contribution of the respective zones. Assuming that the electronic spectra of the conduction and valence bands are described by the dispersion law:

(2) $$E_{1,2}\left(p\right)=\pm {\left[{\left(\frac{\tilde{E_{gs}}}{2}\right)}^{2}+\left(\frac{\tilde{E_{gs}}}{2}\right)p^{2}/m\right]}^{\frac{1}{2}},$$

and the intrinsic defect field represents the value of the Coulomb potential, the energy of the local state has the form [41,42]:

(3) $$E_{i}=\left(\frac{\tilde{E_{gs}}}{2}\right){\left[1-\frac{2me^{4}z^{2}}{\hbar^{2}\xi^{2}\tilde{E_{gs}}}\right]}^{\frac{1}{2}},$$

where, ξ is the permittivity, and the energy is measured from the middle of the band gap. Formula (3) actually corresponds to the two-zone model of the “hydrogen atom” system in the relativistic approximation. The remarkable feature of this result is the non-linear dependence of the depth of the defect level on the band gap (on the surfaces). Therefore, for nanoparticles of different sizes, the depths of the impurity (or defect) levels will necessarily differ, and the stronger they are, the more the radii of these nanoparticles differ (Fermi levels *E_F_*(*R*_1_) ≠ *E_F_*(*R*_2_). This difference immediately leads to the presence of a contact potential:

(4) $$U_{c}\sim f\left(R_{1},R_{2}\right)\cdot \Delta R,$$

Obviously, the origin of such a contact potential is a new mechanism.

In the framework of the two-band impurity model from the general equation of electrical neutrality, the value of the contact potential difference (*U_c_*) for two contacting spherical nanoparticles of different radius (*R*_1_ > *R*_2_), provided that the impurity level is a donor level with energy *ε_D_*, has the form:

(5) $$n_{D}\cdot \frac{4\pi^{2}\hbar^{3}}{A}={\left(2\pi m_{e}k_{0}T\right)}^{\frac{3}{2}}\cdot \left(1+A\cdot \exp\left(\frac{\varepsilon_{D}}{k_{0}T}\right)\right),$$

where *n_D_*—impurity concentration, and $A=exp\left(E_{F}/k_{0}T\right)$.

The solution of the equation for the case of high temperatures has the form:


(6) $$E_{F}=kTln\left(\frac{4n_{D}\pi^{3}\hbar^{3}}{{\left(2\pi m_{e}k_{0}T\right)}^{\frac{3}{2}}}\right),$$

The Fermi level (*E_F_*) does not depend on the nanoparticle radius and

(7) $$U_{c}=E_{F}^{\left(1\right)}-E_{F}^{\left(2\right)}=0,$$

in the case of identical donors, the contact potential difference is zero at high temperatures. In the case of low temperatures, the solution has the form:


(8) $$E_{F}=-\frac{\varepsilon_{D}}{2}+\frac{1}{2}k_{0}T\cdot ln\left(\frac{{\left(2\pi \hbar \right)}^{3}n_{D}}{2{\left(2\pi m_{e}k_{0}T\right)}^{\frac{3}{2}}}\right),$$

The contact potential difference is determined by the dependence of the depth of the donor levels on the radius of the nanoparticles:


(9) $$U_{c}=E_{F}^{\left(1\right)}-E_{F}^{\left(2\right)}=\frac{\left|\varepsilon_{D}\left(R_{1}\right)-\varepsilon_{D}\left(R_{2}\right)\right|}{2},$$

## 5. Conclusions

For the first time, the semiconductor structure in the form of a contact of chemically homogeneous nanoparticles has been practically implemented and studied, which is based on the size effects of band structure distortion that occur when scaling physical objects to a low-dimensional range. 

For the studied systems, a general pattern has been established, the dependence of the type of electrical properties on the moisture concentration in the samples. In particular, it was found that at a low moisture concentration (saturation in a humid atmosphere at a relative humidity of 60% (an island-type layer of water molecules on the surface of nanoparticles) a “False connection” type contact takes place. At 85% atmospheric humidity, the system is saturated (free water in the pores), when objects with different particle sizes come into contact, the contact conductivity changes to a semiconductor type (rectified contact), when objects with the same particle sizes come into contact, linear properties are detected (Ohmic contact). This indicates a significant role of proton transport in the pore volume of the contact of objects on the formation of the electrical continuity of the contact.

For the studied systems, a general regularity has been established, which consists of the extreme nature of the change in the limit electrical properties of contacts depending on the size dispersion and impurity concentration in the material of contacting nanoparticles. It is assumed that the extreme behavior of the electrical properties of the system is due to the concentration constraint on the number of mobile charge carriers with an increase in the particle size and stabilization of their phase composition. It indicates a significant role of the electronic subsystem of nanoparticle material and a dimension of electrical conductivity (2D or 3D) in the formation of the semiconducting properties of the contact.

It has been determined that the difference in particle sizes *Δd* leads to a proportional increase in the reverse voltage, but a decrease in the current. 

The calculated electrical power of the resulting device does not depend significantly on the difference in the sizes of the contacting nanoparticles and, other things being equal, is 4.86 mW for the studied systems. 

Theoretical ideas about the electronic structure near the surface in the case of ionic crystals undergo certain changes. First, for a flat surface, variance of electronic zones is realized, since the band gap on the surface is less than in the bulk, and second, the convexity of the surface of nanoparticles further enhances the variance of electronic zones.

By applying the Keldysh model (1963) to the problem of the depth of the defect or impurity level, it was shown that in ionic-crystal nanoparticles of different sizes, a nonlinear dependence of the depth of the defect level on the radius of the nanoparticle is realized. This leads to the appearance of a contact potential difference.

Of particular note is the question of the nature of peaks on the reverse branch of the *V*-*I* characteristic. It can be assumed that such an unusual pattern is associated with the existence of an inverted jagged potential pit. Indeed, as the reverse voltage increases (the negative electrode is connected to a small nanoparticle), the lower part of the tooth first intersects with the upper boundary of the band gap of the small nanoparticle, and the peak ends with the E boundary reaching the end of the potential pit of the tooth and the transition of electrons from the small nanoparticle to region of the continuous spectrum. This hypothesis needs further analysis.

## Figures and Tables

**Table 1:** Dependence of the Limiting Electrical Parameters of Heterojunctions on Humidity and Size of Contacting Particles.

	Sample	400–400	400–500	400–600	400–700	Humidity, %
Operating Parameter						
Maximum reverse voltage, V		-	-	1.27	2.19	60
Peak reverse current, μA		-	-	−1.36	−1.72	
Maximum forward voltage, V		-	-	−5.59	−4.85	
Peak forward current, μA		-	-	−25.20	−8.49	
Maximum reverse voltage, V		-	0.63	2.93	1.68	75
Peak reverse current, μA		-	1.64	6.21	5.74	
Maximum forward voltage, V		-	−5.36	−5.82	-5.58	
Peak forward current, μA		-	−165.73	−134.42	−132.13	
Maximum reverse voltage, V		0.90	0.78	2.99	2.87	85
Peak reverse current, μA		−1.93	3.46	3.37	4.8	
Maximum forward voltage, V		−5.91	−5.78	−5.85	−5.65	
Peak forward current, μA		−18.51	−262.32	−155.30	−133.32	

**Table 2:** Limit parameters model of curves depending on the percentage of Y_2_O_3_ content.

	Objects	0 mol% Y_2_O_3_	3 mol% Y_2_O_3_	4 mol% Y_2_O_3_	8 mol% Y_2_O_3_	Humidity, %
Objects Operating Parameter						
Maximum reverse voltage, V		-	-	-	-	60
Peak reverse current, μA		-	-	-	-	
Maximum forward voltage, V		-	-	-	-	
Peak forward current, μA		-	-	-	-	
Maximum reverse voltage, V		2.73	0.45	3.29	1.54	75
Peak reverse current, μA		−3.07	0.1	−0.7	−2.32	
Maximum forward voltage, V		−5.67	−5.36	−5.5	−5.41	
Peak forward current, μA		−3.07	−166.96	−62.87	−33.96	
Maximum reverse voltage, V		2.27	0.827	2.62	5.64	85
Peak reverse current, μA		−2.24	3.44	3.44	−3.57	
Maximum forward voltage, V		−6	−5.77	−6	−5.23	
Peak forward current, μA		−8.4	−260.67	−276.08	−93.831	

## Data Availability

Data presented in this article is available on request from the corresponding author.
